# Finding and recognizing objects in natural scenes: complementary computations in the dorsal and ventral visual systems

**DOI:** 10.3389/fncom.2014.00085

**Published:** 2014-08-12

**Authors:** Edmund T. Rolls, Tristan J. Webb

**Affiliations:** ^1^Department of Computer Science, University of WarwickCoventry, UK; ^2^Oxford Centre for Computational NeuroscienceOxford, UK

**Keywords:** object recognition, invariance, saliency, inferior temporal visual cortex, trace learning rule, VisNet

## Abstract

Searching for and recognizing objects in complex natural scenes is implemented by multiple saccades until the eyes reach within the reduced receptive field sizes of inferior temporal cortex (IT) neurons. We analyze and model how the dorsal and ventral visual streams both contribute to this. Saliency detection in the dorsal visual system including area LIP is modeled by graph-based visual saliency, and allows the eyes to fixate potential objects within several degrees. Visual information at the fixated location subtending approximately 9° corresponding to the receptive fields of IT neurons is then passed through a four layer hierarchical model of the ventral cortical visual system, VisNet. We show that VisNet can be trained using a synaptic modification rule with a short-term memory trace of recent neuronal activity to capture both the required view and translation invariances to allow in the model approximately 90% correct object recognition for 4 objects shown in any view across a range of 135° anywhere in a scene. The model was able to generalize correctly within the four trained views and the 25 trained translations. This approach analyses the principles by which complementary computations in the dorsal and ventral visual cortical streams enable objects to be located and recognized in complex natural scenes.

## 1. Introduction

One of the major problems that is solved by the visual system in the cerebral cortex is the building of a representation of visual information that allows object and face recognition to occur relatively independently of size, contrast, spatial frequency, position on the retina, angle of view, lighting, etc. These invariant representations of objects, provided by the inferior temporal visual cortex (Rolls, 2008, 2012), are extremely important for the operation of many other systems in the brain, for if there is an invariant representation, it is possible to learn on a single trial about reward/punishment associations of the object, the place where that object is located, and whether the object has been seen recently, and then to correctly generalize to other views etc. of the same object (Rolls, 2008, 2014). Here we consider how the cerebral cortex solves the major computational task of view-invariant recognition of objects in complex natural scenes, still a major challenge for computer vision approaches, as described in the Discussion.

One mechanism that the brain uses to simplify the task of recognizing objects in complex natural scenes is that the receptive fields of inferior temporal cortex neurons change from approximately 70° in diameter when tested under classical neurophysiology conditions with a single stimulus on a blank screen to as little as a radius of 8° (for a 5° stimulus) when tested in a complex natural scene (Rolls et al., 2003; Aggelopoulos and Rolls, 2005) (with consistent findings described by Sheinberg and Logothetis, 2001). This greatly simplifies the task for the object recognition system, for instead of dealing with the whole scene as in traditional computer vision approaches, the brain processes just a small fixated region of a complex natural scene at any one time, and then the eyes are moved to another part of the screen. During visual search for an object in a complex natural scene, the primate visual system, with its high resolution fovea, therefore keeps moving the eyes until they fall within approximately 8° of the target, and then inferior temporal cortex neurons respond to the target object, and an action can be initiated toward the target, for example to obtain a reward (Rolls et al., 2003). The inferior temporal cortex neurons then respond to the object being fixated with view, size, and rotation invariance (Rolls, 2012), and also need some translation invariance, for the eyes may not be fixating the center of the object when the inferior temporal cortex neurons respond (Rolls et al., 2003).

The questions then arise of how the eyes are guided in a complex natural scene to fixate close to what may be an object; and how close the fixation is to the center of typical objects for this determines how much translation invariance needs to be built into the ventral visual system. It turns out that the dorsal visual system (Ungerleider and Mishkin, 1982; Ungerleider and Haxby, 1994) implements bottom-up saliency mechanisms by guiding saccades to salient stimuli, using properties of the stimulus such as high contrast, color, and visual motion (Miller and Buschman, 2013). (Bottom-up refers to inputs reaching the visual system from the retina). One particular region, the lateral intraparietal cortex (LIP), which is an area in the dorsal visual system, seems to contain saliency maps sensitive to strong sensory inputs (Arcizet et al., 2011). Highly salient, briefly flashed, stimuli capture both behavior and the response of LIP neurons (Bisley and Goldberg, 2003, 2006; Goldberg et al., 2006). Inputs reach LIP via dorsal visual stream areas including area MT, and via V4 in the ventral stream (Soltani and Koch, 2010; Miller and Buschman, 2013). Although top-down attention using biased competition can facilitate the operation of attentional mechanisms, and is a subject of great interest (Desimone and Duncan, 1995; Rolls and Deco, 2002; Deco and Rolls, 2005a; Miller and Buschman, 2013), top-down object-based attention makes only a small contribution to visual search for an object in a complex natural unstructured scene (such as leaves on a tree), increasing the receptive field size from a radius of approximately 7.8 to approximately 9.6° (Rolls et al., 2003), and is not considered further here. Indeed, in these investigations, multiple saccades were required round the scene to find a target object (Rolls et al., 2003).

In the research described here we investigate computationally how a bottom-up saliency mechanism in the dorsal visual stream reaching for example area LIP could operate in conjunction with invariant object recognition performed by the ventral visual stream reaching the inferior temporal visual cortex to provide for invariant object recognition in natural scenes. The hypothesis is that the dorsal visual stream, in conjunction with structures such as the superior colliculus (Knudsen, 2011), uses saliency to guide saccadic eye movements to salient stimuli in large parts of the visual field, and that once a stimulus has been fixated, the ventral visual stream performs invariant object recognition on the region being fixated. The dorsal visual stream in this process knows little about invariant object recognition, so cannot identify objects in natural scenes. Similarly, the ventral visual stream cannot perform the whole process, for it cannot efficiently find possible objects in a large natural scene, because its receptive fields are only approximately 9° in radius in complex natural scenes. It is how the dorsal and ventral streams work together to implement invariant object recognition in natural scenes that we investigate here. By investigating this computationally, we are able to test whether the dorsal visual stream can find objects with sufficient accuracy to enable the ventral visual stream to perform the invariant object recognition. The issue here is that the ventral visual stream has in practice some translation invariance in natural scenes, but this is limited to approximately 9° (Rolls et al., 2003; Aggelopoulos and Rolls, 2005). The computational reason why the ventral visual stream does not compute translation invariant representations over the whole visual field as well as view, size and rotation invariance, is that the computation is too complex. Indeed, it is a problem that has not been fully solved in computer vision systems when they try to perform invariant object recognition over a large natural scene. The brain takes a different approach, of simplifying the problem by fixating on one part of the scene at a time, and solving the somewhat easier problem of invariant representations within a region of approximately 9°.

For this scenario to operate, the ventral visual stream needs then to implement view invariant recognition, but to combine it with some translation invariance, as the fixation position produced by bottom up saliency will not be at the center of an object, and indeed may be considerably displaced from the center of an object. In the model of invariant visual object recognition that we have developed, VisNet, which models the hierarchy of visual areas in the ventral visual stream by using competitive learning to develop feature conjunctions supplemented by a temporal trace or by spatial continuity or both, all previous investigations have explored either view or translation invariance learning, but not both (Rolls, 2012). Combining translation and view invariance learning is a considerable challenge, for the number of transforms becomes the product of the numbers of each transform type, and it is not known how VisNet (or any other biologically plausible approach to invariant object recognition) will perform with the large number, and with the two types of transform combined. Indeed, an important part of the research described here was to investigate how well architectures of the VisNet type generalize between both trained locations and trained views. This is important for setting the numbers of different views and translations of each object that must be trained.

The specific goals of the research and simulations described here were as follows. (1) To demonstrate with a biologically plausible model of the ventral visual system how it could operate to implement view invariant object/person identity recognition with a generic model of the dorsal visual system that produced fixations on parts of scenes that were salient. How would the combined cortical visual areas operate with the dorsal visual system not encoding object identity but only saliency; and the ventral visual system being unable to find objects efficiently in large natural scenes, but able to perform view invariant object recognition once fixation was close to an object? (2) How closely and effectively would a simple, generic, bottom-up saliency system modeling part of the functions of the dorsal visual system find objects in a complex scene, and how accurately would the center of the object be fixated? The accuracy with which the center of the object is fixated is crucial to understand, for this defines how much translation invariance must be incorporated into the ventral visual system for the whole system to work. (3) Can VisNet be trained for both view and translation invariance? This has not been attempted previously with VisNet, and for that matter view invariant object recognition is not a property of most computer vision models (see Discussion). (4) If VisNet can be trained on both view and translation invariant object identification, can it be trained with sufficient translation invariance to cover the visual angle needed given the inaccuracies of the saliency-based fixation mechanism in finding the center of an object, and yet be trained with sufficient views to provide for view-invariant object identification? (5) How well does VisNet generalize from trained views to untrained views of an object? This is important, for it influences how much training of different views is required, which could have an impact on the capacity of the system, that is on the number of objects or people that it can correctly identify with the required translation invariance. (6) How well does VisNet perform in object identification when the objects appear in natural scenes with fixation not necessarily at the trained location, and when views intermediate to those at which VisNet has been trained are presented? That is, how well under the natural scene conditions can VisNet ignore the background and identify a trained object despite it being presented in a view and position that were not trained?

## 2. Methods

### 2.1. Saliency

We chose a bottom up saliency algorithm that is one of the standard ones that has been developed, which adopts the Itti and Koch (2000) approach to visual saliency, and implements it by graph-based visual saliency (GBVS) algorithms (Harel et al., 2006a,b). This system performs well, that is similarly to humans, in many bottom-up saliency tasks. The particular algorithm used for the bottom-up saliency was not crucial to the present research, so we chose a generically representative algorithm^1^. We used static images, so motion was not used to detect saliency. Of course in the human brain, and in a computer application, performance could be made better than described here by using many different cues that can influence saliency, including also color which was disabled in the current algorithm, as VisNet works with grayscale images to help ensure that object shape is being processed, and not a simple feature such as color (Rolls, 2012).

### 2.2. Architecture of the ventral visual stream model, VisNet

The architecture of VisNet has been described previously (Rolls, 2008, 2012), and is summarized briefly next, with a full description provided in the Appendix. Extensions important for the present research included training in both view and translation invariance, together with careful specification of the learning rate during the presentation of each transform, as there were typically 100 or more transforms of every object to be learned.

**Figure 1:**
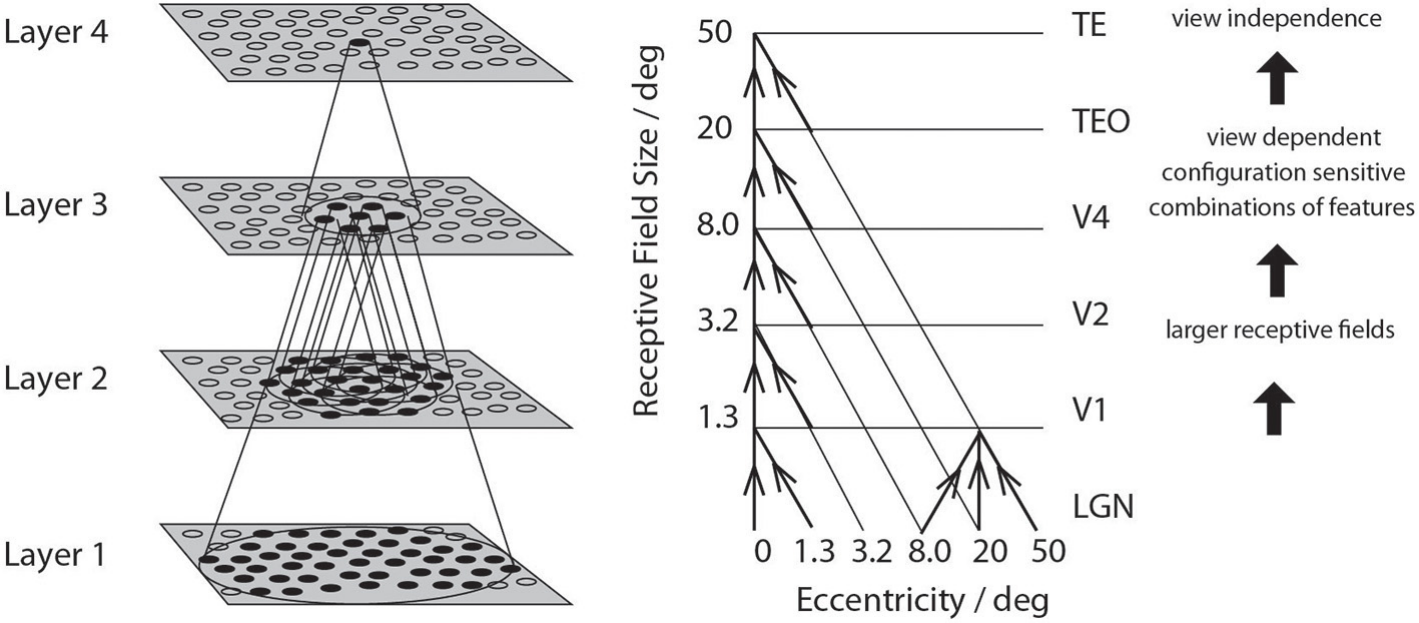
**Convergence in the visual system. Right:** As it occurs in the brain. V1, visual cortex area V1; TEO, posterior inferior temporal cortex; TE, inferior temporal cortex (IT). **Left:** As implemented in VisNet. Convergence through the network is designed to provide fourth layer neurons with information from across the entire input retina.

Fundamental elements of Rolls' 1992 theory for how cortical networks might implement invariant object recognition are described in detail elsewhere (Rolls, 2008, 2012). They provide the basis for the design of VisNet, which can be summarized as:

- A series of competitive networks, organized in hierarchical layers, exhibiting mutual inhibition over a short range within each layer. These networks allow combinations of features or inputs occurring in a given spatial arrangement to be learned by neurons using competitive learning (Rolls, 2008), ensuring that higher order spatial properties of the input stimuli are represented in the network. In VisNet, layer 1 corresponds to V2, layer 2 to V4, layer 3 to posterior inferior temporal visual cortex, and layer 4 to anterior inferior temporal cortex. Layer one is preceded by a simulation of the Gabor-like receptive fields of V1 neurons produced by each image presented to VisNet (Rolls, 2012).
- A convergent series of connections from a localized population of neurons in the preceding layer to each neuron of the following layer, thus allowing the receptive field size of neurons to increase through the visual processing areas or layers, as illustrated in Figure 1.
- A modified associative (Hebb-like) learning rule incorporating a temporal trace of each neuron's previous activity, which, it has been shown (Földiák, 1991; Rolls, 1992; Wallis et al., 1993; Wallis and Rolls, 1997; Rolls and Milward, 2000; Rolls, 2012), enables the neurons to learn transform invariances.

The learning rates for each of the four layers were 0.05, 0.03, 0.005, and 0.005, as these rates were shown to produce convergence of the synaptic weights after 15–50 training epochs. 50 training epochs were run.

The developments to VisNet that facilitated this principled approach to the learning rate, combined view and translation invariance learning, etc, and the parameters used, are described in the Appendix.

### 2.3. Information measures of performance

The performance of VisNet was measured by Shannon information-theoretic measures that are essentially identical to those used to quantify the specificity and selectiveness of the representations provided by neurons in the brain (Rolls and Milward, 2000; Rolls and Treves, 2011; Rolls, 2012). A single cell information measure indicated how much information was conveyed by a single neuron about the most effective stimulus. A multiple cell information measure indicated how much information about every stimulus was conveyed by small populations of neurons, and was used to ensure that all stimuli had some neurons conveying information about them. Details are provided in the Appendix.

### 2.4. Training

**Figure 2:**
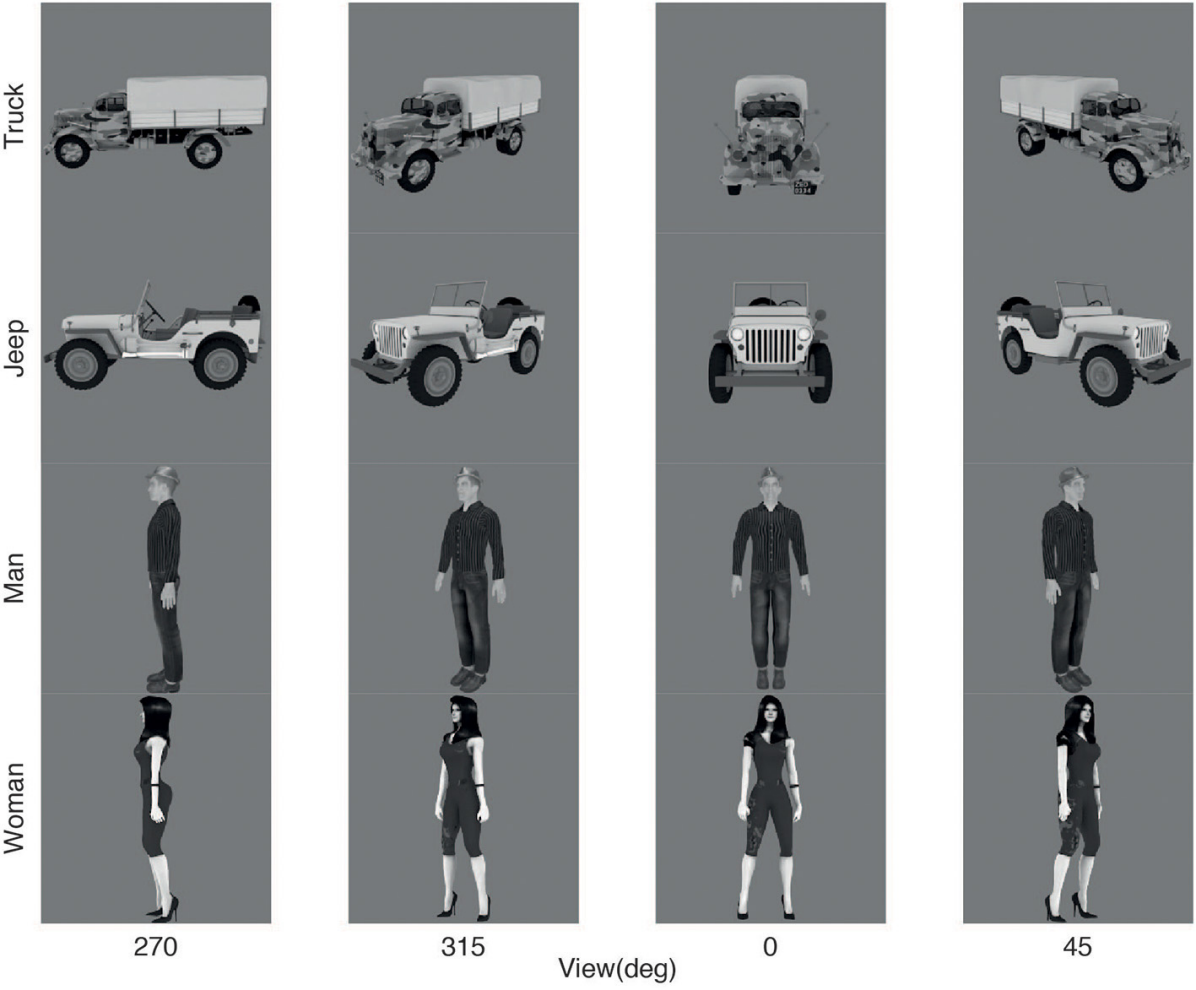
**Training images: 4 views of each of 4 objects**. Each image was 256 × 256 pixels.

VisNet was trained on four views spaced 45° apart of each of the 4 objects as illustrated in Figure 2. The images of each object were generated from a 3D model using Blender (The Blender Foundation, www.blender.org) so that lighting could be carefully controlled. Each grayscale image of an object was 256 × 256 pixels, with the intensity scaled to be in the range 0–255, and the background approximately 127. The object images were pasted into a 512 × 512 gray image to prevent wrap-around effects, prior to the spatial frequency filtering to produce neurons with Gabor-like receptive fields in an emulation of V1 neurons that provided the input to the first layer of VisNet (see Appendix). [We have previously shown that the training need not be on a blank background, provided that the background is not constant across transforms and objects, as will be the case in the natural world (Stringer et al., 2007; Stringer and Rolls, 2008)]

Each training image was trained in 25 locations set out in a 5 × 5 rectangular grid with these locations separated by 8 pixels in the training image. To provide an indication of the range of this translation invariance training, the grid extended between the centers of the headlights in the front view of the jeep shown in Figure 2. This resulted in 100 transforms of each object to be learned. To enable VisNet to learn invariant representations with the trace synaptic learning rule, all the transforms of one object were shown in a random permuted sequence, the trace was reset, and the procedure was repeated with each of the other objects. 50 training epochs were run, as this was sufficient to produce gradual convergence of the synaptic weights over 15–50 epochs, as described in the Appendix.

### 2.5. Testing invariant object recognition in natural scenes

**Figure 3:**
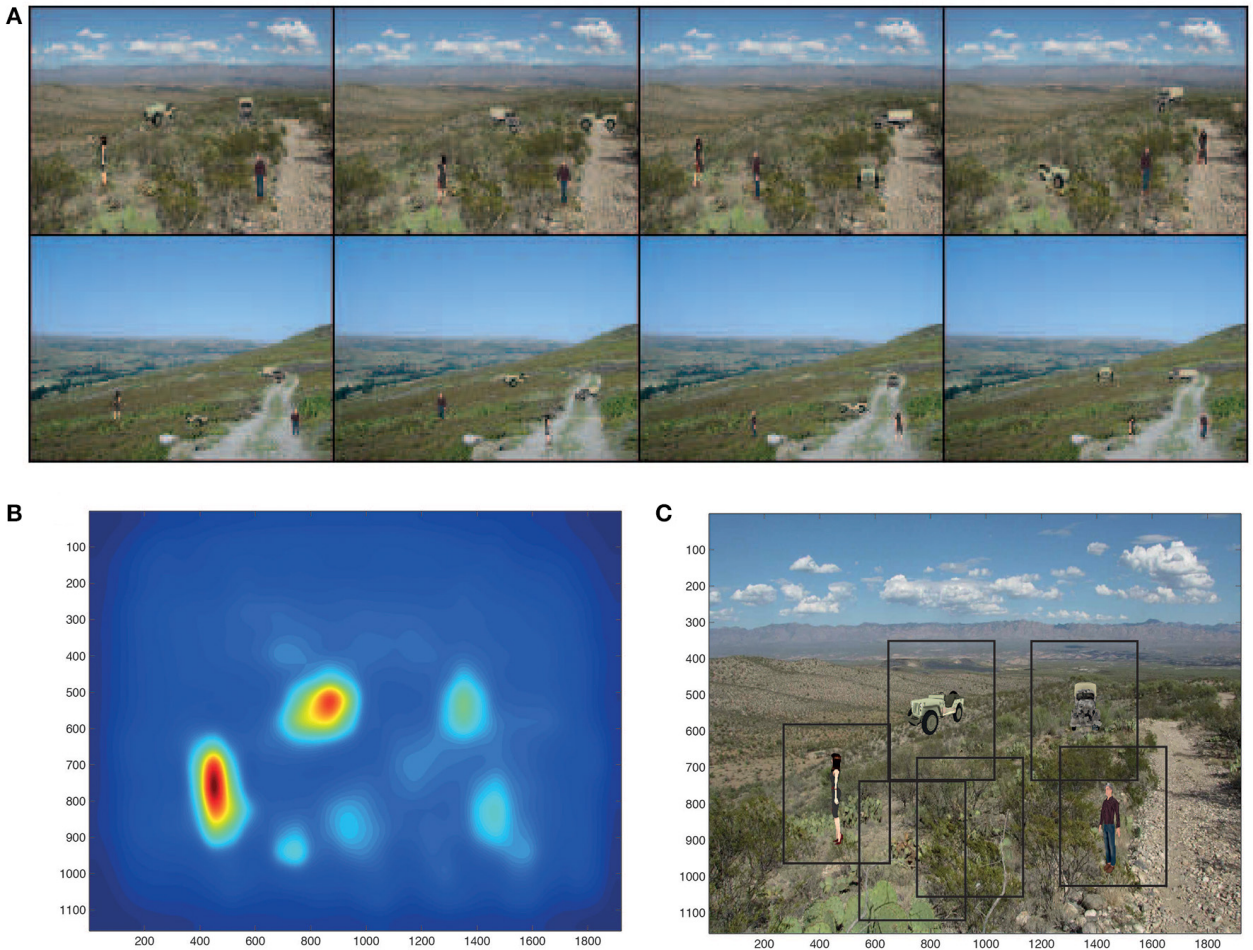
**(A)** Eight of the 12 test scenes. Each scene has 4 objects, each in one of its four views. **(B)** The bottom up saliency map generated by the GBVS code for one of the scenes. The highest levels in the saliency map are red, and the lowest blue. **(C)** Rectangles (384 × 384 pixels) placed around each peak in the scene for which the bottom-up saliency map is illustrated in **(B)**.

Eight of the 12 test scenes are illustrated in Figure 3A. Each scene had each of the objects in one of the four poses. The aim of the combined visual processing was for the dorsal visual stream to detect the salient regions in these 12 scenes, and then for the salient regions to be passed to VisNet to perform the view (and translation) invariant object recognition for every object in the scene. VisNet had been trained on the 4 objects in each of the 4 views, but not on the background scenes, and it was part of the task of VisNet to identify each of the four objects in every scene without being affected by the background clutter of each scene (Stringer and Rolls, 2000). The objects used in this investigation were common types of object with which the human visual system performs good view invariant identification, people and vehicles. Two people and two vehicles were chosen to provide evidence on how the system might operate with typical stimuli for which view-invariant identification is necessary and is performed by the human visual system.

## 3. Results

### 3.1. The operation of the saliency processing

The bottom up saliency map generated by the GBVS code (acting as a surrogate for the dorsal visual system) for one of the scenes is illustrated in Figure 3B. The saliency map has of course no indication of which peak is a trained object, nor of which object it might be.

The saliency maps generated by GBVS correspond closely to the saccades and resulting fixations of humans (Itti and Koch, 2000; Harel et al., 2006a,b). We therefore extracted images from the scene that were at the center of each peak of the saliency map. A weighted centroid was used, as implemented in MATLAB. Each extracted image centered on a peak in the saliency map was 384 × 384 pixels (not the originally trained 256 × 256 size of a training image), because sometimes a saliency peak was not well centered on an object, and we wished to be sure that the whole object was in the image presented to VisNet. Figure 3C shows rectangles produced in this way round the 6 most salient regions in the test scene for which the saliency map is shown in Figure 3B. Four of the saliency peaks and therefore the rectangles contained trained objects, and two extracted images just salient parts of the background scene in which the trained objects appeared.

The extracted (“foveated”) images of the objects to be presented to VisNet based on saliency are not always well-centered in the 384 × 384 extracted image, and this is clear for one of the objects, the man, as shown in Figure 3C.

**Figure 4:**
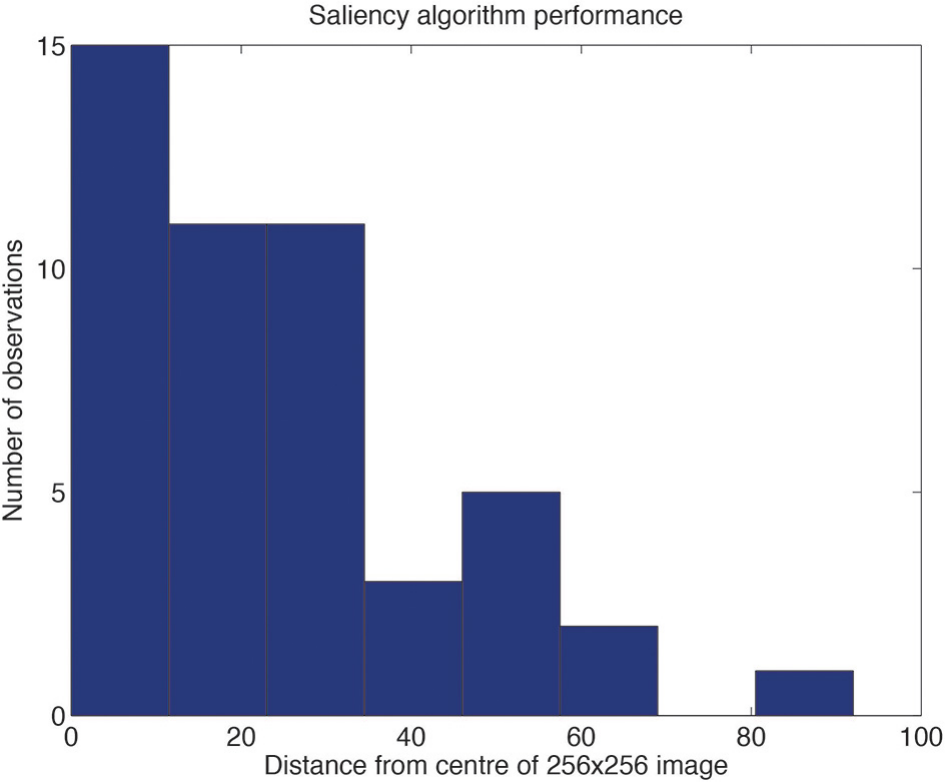
**Distribution of the offsets of the saliency peaks from the center of each object**. The data were obtained for 48 images (different views of the different objects) presented in 3 backgrounds. An example of one of the backgrounds containing one view of each of four objects is illustrated in Figure 3C.

To provide evidence on the degree of translation invariance that would be required of VisNet given that the center of each image was not always at the peak of the saliency map, so that the extracted image would be offset from a central trained location, the offsets of the saliency peaks from the center of each object image are shown in Figure 4. While it is clear that the majority of the offsets of the saliency peak from the center of the object were in the range 0–32 pixels, some were beyond this. For this reason, we do not necessarily expect that VisNet, trained on a grid with an offset up to 32 would achieve 100% correct object recognition. The evidence shown in Figure 4 does provide though the useful indication that training to allow for offsets up to 64 for a 256 × 256 image might improve performance.

### 3.2. Tests of VisNet on view and translation invariance

**Figure 5:**
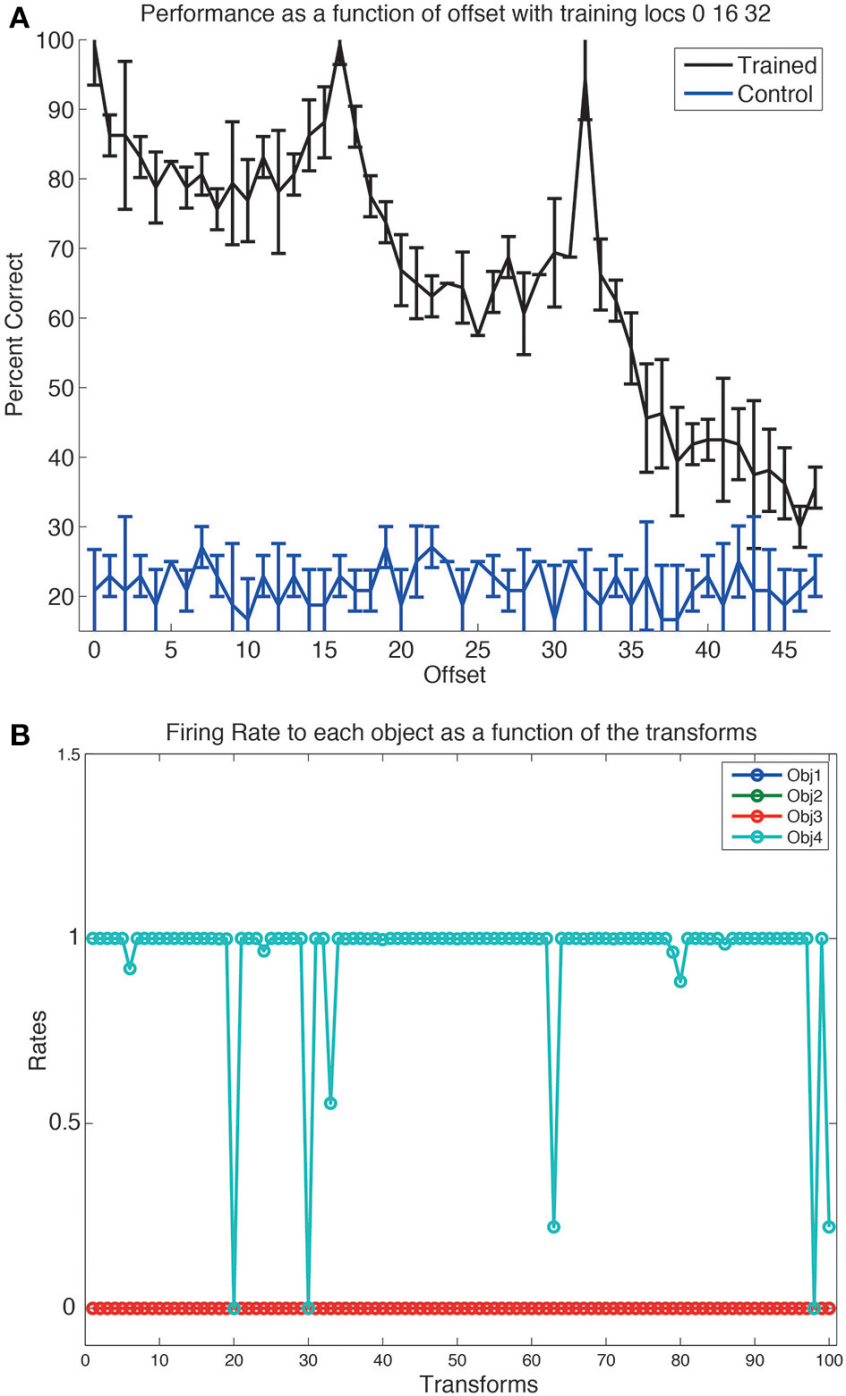
**(A)** The performance on the view invariant object recognition tested with images at the 15 trained locations on the 64 × 64 training grid, and at intermediate locations. The ordinate shows the distance from the central line in the training grid, and trained locations thus correspond to offsets of 0, 16, and 32. The mean and standard deviation are shown for each data point. The standard deviation was measured by performing the training ten times each with a different random seed to generate the connectivity of VisNet. Performance decreases beyond an offset of 32, because there was no translation invariant training beyond this. **(B)** A neuron in layer 4 of VisNet that responded to almost all transforms of one object (4), and to no transform of any other object (1–3). There were 25 location transforms on a grid of size 64 with a spacing of 16, and 4 views of each object at each location. The stimulus-specific information or surprise was 2 bits, as there were 4 objects.

Although VisNet had been trained on a 25-location grid with size 64 × 64 with spacing of 16 pixels, and with 4 different views of each object, we did not know how well VisNet would perform on this task as this has never been tested before, nor whether performance would generalize to intermediate locations in the 64 × 64 grid, given that there were only 25 training locations spaced 16 pixels apart. An analysis is shown in Figure 5A which covers the 4096 locations in the 64 × 64 grid. This indicates that the performance (on the view invariant object recognition) peaks at the trained locations (0, 16, and 32 in this Figure), but also that there is reasonable performance at intermediate locations between the training locations. (The chance performance with 4 objects is 25% correct.) This is an important new result, which adds to previous evidence that smaller versions of VisNet with 32 × 32 neurons in each of 4 layers can generalize reasonably across intermediate untrained locations in scenes with blank backgrounds (Wallis and Rolls, 1997). The performance was measured with a pattern associator trained on layer 4 of VisNet, with four output neurons (one for each object), and the 25 most selective cells for each object identified using the single cell information measure (see Appendix). The best cells were quite selective for one of the objects, and quite invariant in their response over the 100 transforms (4 views and 25 locations), as illustrated in Figure 5B.

### 3.3. Tests of the whole saliency plus view invariance system

With 48 images extracted from the the 12 test scenes (8 illustrated in Figure 3A), performance was 90% correct (43 correct/48), where chance with the four objects is 25% (Fisher test *p* « 0.0001).

It is important that this good performance on this identification task was found when the images extracted for presentation to VisNet had background parts of the scene included (e.g., Figure 3C). These background features did not produce large decreases in the performance of VisNet, given that VisNet had been trained on the objects but not on the backgrounds (Stringer and Rolls, 2000). This is important for the processes of invariant visual object identification in novel complex natural scenes described here. Further, if there was a low amplitude saliency peak containing only part of the background scene and not an object, then VisNet did not respond to this as a trained object. When errors were made by VisNet on the object identification, the confusions were as frequent between the classes of people and vehicle as within these classes.

### 3.4. Tests of view plus translation invariance at intermediate views

**Figure 6:**
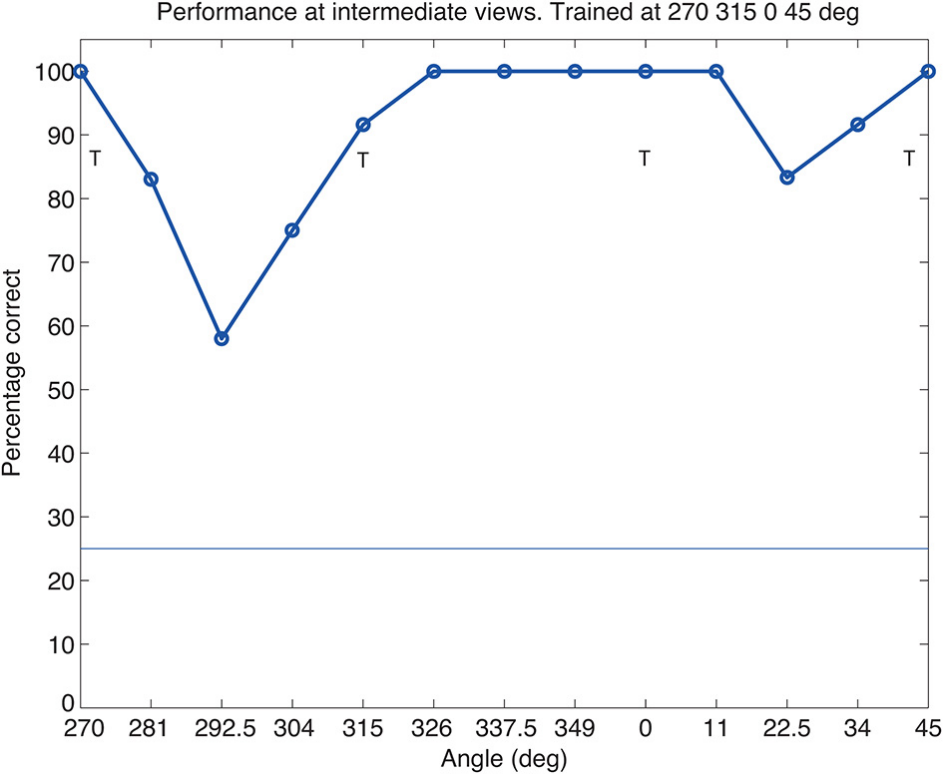
**Performance of VisNet at views intermediate to the trained views of 270, 315, 0, and 45°, which are indicated by T**. Performance was tested at 6 intermediate views between each trained view, and then for illustrative purposes the results for the 6 intermediate views were averaged using adjacent views. Each data point shown is the average of 12 observations. The chance level of performance, 25%, is indicated.

The training images had four views of each object separated by 45° as illustrated in Figure 2. To assess whether these views were sufficiently close to allow for generalization between the trained views, we tested VisNet with 6 intermediate views (presented on plain backgrounds) between each trained view. As shown in Figure 6, performance is reasonable at the untrained intermediate views. The important implication is that VisNet does not need to be trained on a large set of closely spaced views, and this helps the rapid learning of new objects, and also may help to increase the capacity of VisNet, as only few views of each new object need to be learned.

## 4. Discussion

By combining in a simulation the operation of the dorsal and ventral visual systems in the identification of objects in complex natural scenes, we believe that important progress has been made, in a biologically inspired approach not attempted in other including computer-based approaches. The models simulated show how the brain may solve this major computational problem by moving the eyes to fixate close to objects in a natural scene using bottom-up saliency implemented in the dorsal visual system, and then performs objects recognition successively for each of the fixated regions using the ventral visual system. The research described here emphasizes that because the eyes do not locate the center of objects based on saliency, then translation invariance as well as view, size etc invariance needs to be implemented in the ventral visual system. We show how a model of invariant object recognition in the ventral visual system, VisNet, can perform the required combination of translation and view invariant recognition, and moreover can generalize between views of objects that are 45° apart during training, and can also generalize to intermediate locations when trained in a coarse training grid with the spacing between trained locations equivalent to 1–3°.

We emphasize that the model is closely linked to neurophysiological research on visual object recognition in natural scenes, and explicitly models how the system could operate computationally to achieve the degree of translation invariance shown in complex natural scenes by inferior temporal cortex neurons (Rolls et al., 2003; Aggelopoulos and Rolls, 2005) as well as the view invariance that is combined with this (Hasselmo et al., 1989; Booth and Rolls, 1998). Moreover, the deformation or pose invariance that can be shown by inferior temporal cortex neurons is also a property that can be learned by this functional architectural computational model of object recognition in the ventral visual system, VisNet (Webb and Rolls, 2014).

We note that in the underlying neurophysiological experiments, the objects were small and were presented in an unstructured scene, which was the leaves of trees (Rolls et al., 2003). In this type of scene, objects can only be found by repeated saccades round the scene until the eyes become sufficiently close for the object to fall within the inferior temporal visual cortex neuronal receptive fields which become dynamically reduced to a few degrees in such scenes (Rolls et al., 2003). The receptive fields of inferior temporal cortex neurons are thus small, a few degrees, in complex natural scenes (Rolls et al., 2003; Aggelopoulos and Rolls, 2005). In previous research, sometimes large receptive fields have been reported (Gross et al., 1969), and sometimes small, a few degrees (Op de Beeck and Vogels, 2000; DiCarlo and Maunsell, 2003). We showed that an important factor in the receptive field size is the background. If the receptive fields are measured as in traditional visual neurophysiology against a blank background, then the receptive fields can be as large as 70°, whereas in a complex cluttered natural scene the receptive fields can be as small as a few degrees (Rolls et al., 2003). Moreover, we went on to show that the underlying dynamical mechanism for receptive field size adjustment is probably competition between neurons operating with neurons that have more input from objects close to the fovea (Trappenberg et al., 2002). If objects can be recognized by humans rapidly without the need for multiple fixations round the scene (Thorpe, 2009), then one has to assume that the scene has properties including probably some structure or contrast or color or other low-level feature (Crouzet and Thorpe, 2011), that enables the object to pop out using lower-level processing that does not engage the invariant representations provided by inferior temporal cortex neurons (Rolls, 2012).

The operation of VisNet coupled with the saliency model of the dorsal visual system described here for the identification of multiple objects at different positions in a natural scene with view invariance is now compared with that of other systems and approaches. First, VisNet provides a theory and model of how object identification with view (Stringer and Rolls, 2002), size (Wallis and Rolls, 1997), isomorphic rotation, translation (Stringer and Rolls, 2000; Perry et al., 2010), contrast, illumination (Rolls and Stringer, 2006), and spatial frequency invariance is performed in the cerebral cortex (Rolls, 2012). The approach is addressing fundamental issues about how the cerebral cortex functions. VisNet models four stages of visual processing beyond V1, and simulates V1; it uses local, biologically plausible, synaptic learning rules; it produces neurons in its layer 4 that are comparable to neurons recorded in the inferior temporal visual cortex (IT) (Rolls and Treves, 2011; Rolls, 2012) in terms of their receptive fields and how they are influenced by multiple items in a scene and by top-down attention (Trappenberg et al., 2002; Rolls et al., 2003); in terms of the neuronal tuning to different objects (though VisNet has somewhat more binary neurons that IT neurons) (Rolls, 2008, 2012; Rolls and Treves, 2011); and in terms of size, view, translation, spatial frequency, and contrast invariance (Rolls, 2012). We know of no other biologically plausible model that performs view invariant as well as other types of transform invariant object identification, and that can do this with multiple different objects in complex natural scenes, as demonstrated here.

We provide now (following a suggestion) an account of how VisNet is able to solve the type of invariant object recognition problem described here when an image is presented to it, with more detailed accounts available elsewhere (Wallis and Rolls, 1997; Rolls, 2008, 2012). VisNet is a 4-layer network with feedforward convergence from stage to stage that enables the small receptive fields present in its V1-like Gabor filter inputs of approximately 1° to increase in size so that by the fourth layer a single neuron can potentially receive input from all parts of the input space (Figure 1). The feedforward connections between layers are trained by competitive learning, which is an unsupervised form of learning (Rolls, 2008), that allows neurons to learn to respond to feature combinations. As one proceeds up though the hierarchy, the feature combinations become combinations of feature combinations (see Rolls, 2008 Figure 4.20 and Elliffe et al., 2002). Local lateral inhibition within each layer allows each local area within a layer to respond to and learn whatever is present in that local region independently of how much information and contrast there may be in other parts of a layer, and this, together with the non-linear activation function of the neurons, enables a sparse distributed representation to be produced. In the sparse distributed representation, a small proportion of neurons is active at a high rate for the input being presented, and most of the neurons are close to their spontaneous rate, and this makes the neurons of VisNet (Rolls, 2008, 2012) very similar to those recorded in the visual system (Rolls, 2008; Rolls and Treves, 2011). A key property of VisNet is the way that it learns whatever can be learned at every stage of the network that is invariant as an image transforms in the natural world, using the temporal trace learning rule. This learning rule enables the firing from the preceding few items to be maintained, and given the temporal statistics of visual inputs, these inputs are likely to be from the same object. (Typically primates including humans look at one object for a short period during which it may transform by translation, size, isomorphic rotation, and/or view, and all these types of transform can therefore be learned by VisNet.) Effectively, VisNet uses as a teacher the temporal and spatial continuity of objects as they transform in the world to learn invariant representations. (An interesting example is that representations of individual people or objects invariant with respect to pose (e.g., standing, sitting, walking) can be learned by VisNet, or representations of pose invariant with respect to the individual person or object can be learned by VisNet depending on the order in which the identical images are presented during training Webb and Rolls, 2014.) Indeed, we developed these hypotheses (Rolls, 1992, 1995, 2012; Wallis et al., 1993) into a model of the ventral visual system that can account for translation, size, view, lighting, and rotation invariance (Wallis and Rolls, 1997; Rolls and Milward, 2000; Stringer and Rolls, 2000, 2002, 2008; Rolls and Stringer, 2001, 2006, 2007; Elliffe et al., 2002; Perry et al., 2006, 2010; Stringer et al., 2006, 2007; Rolls, 2008, 2012). Consistent with the hypothesis, we have demonstrated these types of invariance (and spatial frequency invariance) in the responses of neurons in the macaque inferior temporal visual cortex (Rolls et al., 1985, 1987, 2003; Rolls and Baylis, 1986; Hasselmo et al., 1989; Tovee et al., 1994; Booth and Rolls, 1998). Moreover, we have tested the hypothesis by placing small 3D objects in the macaque's home environment, and showing that in the absence of any specific rewards being delivered, this type of visual experience in which objects can be seen from different views as they transform continuously in time to reveal different views leads to single neurons in the inferior temporal visual cortex that respond to individual objects from any one of several different views, demonstrating the development of view-invariance learning (Booth and Rolls, 1998). (In control experiments, view invariant representations were not found for objects that had not been viewed in this way.) The learning shown by neurons in the inferior temporal visual cortex can take just a small number of trials (Rolls et al., 1989). The finding that temporal contiguity in the absence of reward is sufficient to lead to view invariant object representations in the inferior temporal visual cortex has been confirmed (Li and DiCarlo, 2008, 2010, 2012). The importance of temporal continuity in learning invariant representations has also been demonstrated in human psychophysics experiments (Perry et al., 2006; Wallis, 2013). Some other simulation models are also adopting the use of temporal continuity as a guiding principle for developing invariant representations by learning (Wiskott and Sejnowski, 2002; Wiskott, 2003; Wyss et al., 2006; Franzius et al., 2007), and the temporal trace learning principle has also been applied recently (Isik et al., 2012) to HMAX (Riesenhuber and Poggio, 2000; Serre et al., 2007c).

We now compare this VisNet approach to invariant object recognition to some other approaches that seek to be biologically plausible. One such approach is HMAX (Riesenhuber and Poggio, 2000; Serre et al., 2007a,b,c; Mutch and Lowe, 2008), which is a hierarchical feedforward network with alternating simple cell-like (S) and complex cell-like (C) layers. The simple cell-like layers respond to a similarity function of the firing rates of the input neuron to the synaptic weights of the receiving neuron (used as an alternative to the more usual dot product), and the complex cells to the maximum input that they receive from a particular class of simple cell in the preceding layer. The classes of simple cell are set to respond maximally to a random patch of a training image (by presenting the image, and setting the synaptic weights of the S cells to be the firing rates of the cells from it receives), and are propagated laterally, that is there are exact copies throughout a layer, which is of course a non-local operation and not biologically plausible. The hierarchy receives inputs from Gabor-like filters (which is like VisNet). The result of this in HMAX is that in the hierarchy there is no learning of invariant representations of objects; and that the output firing in the final C layer (for example the second C layer in a four-layer S1-C1-S2-C2 hierarchy) is high for almost all neurons to most stimuli, with almost no invariance represented in the output layer of the hierarchy, in that two different views of the same object may be as different as a view of another object, measured using the responses of a single neuron or of all the neurons (Robinson and Rolls, 2014). The neurons in the output C layer are thus quite unlike those in VisNet or in the inferior temporal cortex, where there is a sparse distributed representation, and where single cells convey much information in their firing rates, and populations of single cells convey much information that can be decoded by biologically plausible dot product decoding such as might be performed by a pattern association network in the areas that receive from the inferior temporal visual cortex, such as the orbitofrontal cortex and amygdala (Rolls, 2008, 2012; Rolls and Treves, 2011). HMAX therefore must resort to a very powerful classification algorithm, in practice typically a Support Vector Machine (SVM), which is not biologically plausible, to learn to classify all the outputs of the final layer that are produced by the different transforms of one object to be of the same object, and different to those of other objects. Thus HMAX does not learn invariant representations by its output layer of the S–C hierarchy, but instead uses a SVM to perform the classification that the SVM is taught. This is completely unlike the output of VisNet and of inferior temporal cortex neuron firing, which by responding very similarly in terms of firing rate to the different transforms of an object show that the invariance has been learned in the hierarchy (Rolls, 2008, 2012). Another way that the output of HMAX may be assessed is by the use of View-Tuned Units (VTUs), each of which is set to respond to one view of a class or object by setting its synaptic weights from each C unit to the value of the firing of the C unit to one view or exemplar of the object or class (Serre et al., 2007b). Because there is little invariance in the C units, many different VTUs are needed, with one for each training view or exemplar. Because the VTUs are different to each other for the different views of the same object or class, a further stage of training is then needed to classify the VTUs into object classes, and the type of learning is least squares error minimization (Serre et al., 2007b), equivalent to a delta-rule one-layer perceptron which again is not biologically plausible for neocortex (Rolls, 2008). Thus HMAX does not generate invariant representations in its S–C hierarchy, and in the VTU approach uses two layers of learning after the S–C hierarchy, the second involving least squares learning, to produce classification. This is unlike VisNet, which learns invariant representations in its hierarchy, and produces view invariant neurons (similar to those for faces (Hasselmo et al., 1989) and objects (Booth and Rolls, 1998) in the inferior temporal visual cortex) that can be read by a biologically plausible pattern associator (Rolls, 2008, 2012).

Another difference of HMAX from VisNet is in the way that VisNet is trained, which is a fundamental aspect of the VisNet approach. HMAX has traditionally been tested with benchmarking databases such as the CalTech-101 and CalTech-256 (Griffin et al., 2007) in which sets of images from different categories are to be classified. The Caltech-256 dataset is comprised of 256 object classes made up of images that have many aspect ratios, sizes and differ quite significantly in quality (having being manually collated from web searches). The objects within the images show significant intra-class variation and have a variety of poses, illumination, scale and occlusion as expected from natural images. A network is supposed to classify these correctly into classes such as hats and bears (Rolls, 2012; Robinson and Rolls, 2014). The problem is that examples of each class of object transforming continuously though different positions on the retina, size, isomorphic rotation, and view are not provided to help the system learn about how a given type of object transforms in the world. The system just has to try to classify based on a set of often quite different exemplars that are not transforms of each other. Thus a system trained in this way is greatly hindered in generating transform invariant representations by the end of the hierarchy, and such a system has to rely on a powerful classifier such as a SVM to perform a classification that is not based on transform invariance learned in the hierarchical network. In contrast, VisNet is provided during training with systematic transforms of objects of the type that would be seen as objects transform in the world, and has a well-posed basis for learning invariant representations. It is important that with VisNet, the early layers may learn what types of transform can be produced in small parts of the visual field by different classes of object, so that when a new class of object is introduced, rapid learning in the last layer and generalization to untrained views can occur without the need for further training of the early layers (Stringer and Rolls, 2002).

Some other approaches to biologically plausible invariant object recognition are being developed with hierarchies that may be allowed unsupervised learning (Pinto et al., 2009; DiCarlo et al., 2012; Yamins et al., 2014). For example, a hierarchical network has been trained with unsupervised learning, and with many transforms of each object to help the system to learn invariant representations in an analogous way to that in which VisNet is trained, but the details of the network architecture are selected by finding parameter values for the specification of the network structure that produce good results on a benchmark classification task (Pinto et al., 2009). However, formally these are convolutional networks, so that the neuronal filters for one local region are replicated over the whole of visual space, which is computationally efficient but biologically implausible. Further, a general linear model is used to decode the firing in the output level of the model to assess performance, so it is not clear whether the firing rate representations of objects in the output layer of the model are very similar to that of the inferior temporal visual cortex. In contrast, with VisNet (Rolls and Milward, 2000; Rolls, 2012) the information measurement procedures that we use (Rolls et al., 1997a,b) are the same as those used to measure the representation that is present in the inferior temporal visual cortex (Tovee et al., 1993; Rolls and Tovee, 1995; Tovee and Rolls, 1995; Abbott et al., 1996; Baddeley et al., 1997; Rolls et al., 1997a,b, 2004, 2006; Panzeri et al., 1999; Treves et al., 1999; Franco et al., 2004, 2007; Aggelopoulos et al., 2005; Rolls and Treves, 2011).

We turn next to compare the operation of VisNet, as a model of cerebral cortical mechanisms involved in view-invariant object identification, with artificial, computer vision, approaches to object identification. However, we do emphasize that our aim in the present research is to investigate how the cerebral cortex operates in vision, not how computer vision attempts to solve similar problems. Within computer vision, we note that many approaches start with using independent component analysis (ICA) (Kanan, 2013), sparse coding (Kanan and Cottrell, 2010), and other mathematical approaches (Larochelle and Hinton, 2010) to derive what may be suitable “feature analyzers,” which are frequently compared to the responses of V1 neurons. Computer vision approaches to object identification then may take combinations of these feature analyzers, and perform statistical analyses using computer-based algorithms that are not biologically plausible such as Restricted Boltzmann Machines (RBMs) on these primitives to statistically discriminate different objects (Larochelle and Hinton, 2010). Such a system does not learn view invariant object recognition, for the different views of an object may have completely different statistics of the visual primitives, yet are the different views of the same object. (Examples might include frontal and profile views of faces, which are well tolerated for individual recognition by some inferior temporal cortex neurons (Hasselmo et al., 1989); very different views of 3D object which are identified correctly as the same object by IT neurons after visual experience with the objects to allow for view-invariant learning (Booth and Rolls, 1998); and many man-made tools and objects which may appear quite different in 2D image properties from different views.) Part of the difficulty of computer vision lay in attempts to parse a whole scene at one time (Marr, 1982). However, the biological approach is to place the fovea on one part of a scene, perform image analysis/object identification there, and then move the eyes to fixate a different location in a scene (Trappenberg et al., 2002; Rolls et al., 2003). This is a divide-and-conquer strategy used by the real visual system, to simplify the computational problem into smaller parts performed successively, to simplify the representation of multiple objects in a scene, and to facilitate passing the coordinates of a target object for action by using the coordinates of the object being fixated (Ballard, 1990; Rolls and Deco, 2002; Rolls et al., 2003; Aggelopoulos and Rolls, 2005; Rolls, 2008, 2012). This approach has now been adopted by some computer vision approaches (Denil et al., 2012).

Important issues are raised for future research.

First, how well does this approach scale up? At present there are 128 × 128 neurons in each of 4 layers of VisNet, that is 65,536 neurons. This is small compared to the number of neurons in the ventral visual stream, which number tens of millions of neurons (Rolls, 2008). If this is indeed a good model of the processing in the ventral visual system, as we hypothesize and on which VisNet is based (Rolls, 2012), then the system should scale up appropriately, that is, probably linearly. There are a number of different aspects that need to scale up. One is the number of objects that can be trained. A second is the number of views that can be trained. A third is the number of locations in which the system is trained, both because saliency mechanisms are not as accurate as the range of 32 pixels from the fovea over which we trained here (Figure 4), and because it may be advantageous to train at intermediate locations (Figure 5). We propose to scale up VisNet by 16 times, from 128 × 128 neurons per layer to 512 × 512 neurons per layer, and to simultaneously address all these issues.

Second, we have used a generically sound and well-known approach to bottom-up saliency, an approach developed by Koch, Itti, Harel and colleagues (Itti and Koch, 2000; Harel et al., 2006a,b). However, it is possible to tune saliency algorithms so that they are more likely to detect objects of certain classes, such as faces or cars. This may greatly increase the capability of the approach described here, and we plan to test how much improvement in performance for the detection and then identification of certain classes of objects can be obtained by incorporating more specialized saliency algorithms. Many saliency approaches and algorithms that are of interest for future research are available (Bruce and Tsotsos, 2006; Achanta et al., 2008; Zhang et al., 2008; Kootstra et al., 2010; Goferman et al., 2012; Riche et al., 2012; Jia et al., 2013; Li et al., 2013). For example, contextual information may be useful, such as the fact that sofas are not usually found in the sky, and that people are usually tall, skinny objects on the ground (though see Webb and Rolls, 2014), and contextual guidance models have been combined with bottom-up saliency models (Oliva and Torralba, 2006; Torralba et al., 2006; Ehinger et al., 2009; Kanan et al., 2009). We emphasize that in the system described here, only one fixation is assumed for each object in a scene, consistent with the fact that single neurons in the inferior temporal visual cortex provide sufficient information for object and face identification during a single fixation and in only 20–50 ms of neuronal firing, as shown by information theoretic analyses of neuronal activity and by backward masking (Rolls et al., 1994; Rolls and Tovee, 1994; Tovee and Rolls, 1995). [More detailed information may become available with repeated fixations on different parts of an object, and this has been investigated in computer vision (Barrington et al., 2008; Kanan and Cottrell, 2010; Larochelle and Hinton, 2010).]

Third, we have not utilized top-down attention in the developments described here. Top-down attention, whereby an object or set of objects is held active in a short term memory which biases the competitive networks in VisNet, can in principle improve performance considerably (Rolls and Deco, 2002; Deco and Rolls, 2005b; Rolls, 2008). Indeed, we have developed and successfully tested a reduced version of VisNet in which top-down attention does facilitate processing (Deco and Rolls, 2004), and this approach has also been used in computer vision (Walther et al., 2002). Another type of top-down effect is that task requirements can influence fixations in a scene (Hayhoe and Ballard, 2005). We plan in future to incorporate top-down attention into the full, current, version of VisNet, to investigate how this is likely to improve performance, especially for certain selected classes of object.

Fourth, it will be useful to investigate in future the incorporation of more powerful synaptic learning rules when training with the large number of transforms needed when learning invariance for both view and translation transforms of objects. With VisNet, we have so far used an associative (Hebbian) synaptic modification rule (with a trace of previous firing in the postsynaptic term), for biological plausibility (Rolls, 2012). However, to explore further the potential of the overall architecture of VisNet, it will be of interest to investigate how much performance improves when error correction of the post-synaptic firing with respect to the trace of previous neuronal activity is incorporated to implement gradient descent. Gradient descent (Einhauser et al., 2005; Wyss et al., 2006) or optimized slow learning (Wiskott and Sejnowski, 2002; Wiskott, 2003) have been found useful with different architectures.

Fifth, if a strong saliency peak occurs due to something in the background scene that is close to an object, or due to another trained object, how will the system respond? We suggest that the general answer is that the asymmetry that is present in the receptive fields of inferior temporal cortex neurons in cluttered scenes (Aggelopoulos and Rolls, 2005) that is related to the asymmetries caused by the sparse probabilistic forward connections of each neuron (Rolls et al., 2008) and that enables two instances of the same object close together to be correctly identified in terms of both object and position (Rolls et al., 2008) provides the solution, but it will be of interest to investigate this in detail.

Part of the value of the research described here is that it tests, and investigates the operation of, a theory of how view invariant object identification could be implemented by the cerebral cortex. Some predictions of the simulations are (1) that learning will need to be part of the process involved in view-invariant object identification, as the views of an object can be very different; (2) that for at least views of people, a few well-spaced views (we used 45°) should suffice; (3) that translation invariance in complex unstructured crowded scenes may need to be over just a few degrees, for fixation guided by bottom-up saliency has precision of that order at least for the types of object considered here, and repeated saccades are necessary to reach sufficiently close to an object in a large scene for the invariance available to be able to operate in object identification (Rolls et al., 2003; Aggelopoulos and Rolls, 2005); and (4) that just a single fixation of each object will in general suffice for object/person identification, because of the speed of cortical processing (Rolls and Treves, 2011; Rolls, 2012).

### Conflict of interest statement

The authors declare that the research was conducted in the absence of any commercial or financial relationships that could be construed as a potential conflict of interest.

## Footnotes

^1^GBVS was used with its default parameters, except as follows: channels = CIO; gaborangles 0, 30, 60, 90, 120, 150; onCenterBias = 1; levels 2 3; sigma_frac_act = 0.35; sigma_frac_norm = 0.26.

## A. Appendix: the architecture of VisNet

This Appendix describes the functional architecture, operation, and testing of VisNet as used in this paper. VisNet is a hierarchical feedforward 4-layer network that models properties of the ventral visual system involved in invariant visual object recognition (Rolls, 2008, 2012).

### A.1 The trace rule

The learning rule implemented in the VisNet simulations utilizes the spatio-temporal constraints placed upon the behavior of “real-world” objects to learn about natural object transformations. By presenting consistent sequences of transforming objects the cells in the network can learn to respond to the same object through all of its naturally transformed states, as described by Földiák (1991), Rolls (1992), Wallis et al. (1993), Wallis and Rolls (1997), and Rolls (2012). The learning rule incorporates a decaying trace of previous cell activity and is henceforth referred to simply as the “trace” learning rule. The learning paradigm we describe here is intended in principle to enable learning of any of the transforms tolerated by inferior temporal cortex neurons, including position, size, view, lighting, and spatial frequency (Rolls, 1992, 2000; Rolls and Deco, 2002; Rolls, 2008, 2012).

Various biological bases for this temporal trace have been advanced as follows: [The precise mechanisms involved may alter the precise form of the trace rule which should be used. Földiák (1992) describes an alternative trace rule which models individual NMDA channels. Equally, a trace implemented by temporally extended cell firing in a local cortical attractor could implement a short-term memory of previous neuronal firing (Rolls, 2008).]

- The persistent firing of neurons for as long as 100–400 ms observed after presentations of stimuli for 16 ms (Rolls and Tovee, 1994) could provide a time window within which to associate subsequent images. Maintained activity may potentially be implemented by recurrent connections between as well as within cortical areas (Rolls and Treves, 1998; Rolls and Deco, 2002; Rolls, 2008). [The prolonged firing of inferior temporal cortex neurons during memory delay periods of several seconds, and associative links reported to develop between stimuli presented several seconds apart (Miyashita, 1988) are on too long a time scale to be immediately relevant to the present theory. In fact, associations between visual events occurring several seconds apart would, under *normal* environmental conditions, be detrimental to the operation of a network of the type described here, because they would probably arise from different objects. In contrast, the system described benefits from associations between visual events which occur close in time (typically within 1 s), as they are likely to be from the same object.]
- The binding period of glutamate in the NMDA channels, which may last for 100 ms or more, may implement a trace rule by producing a narrow time window over which the *average* activity at each presynaptic site affects learning (Földiák, 1992; Rolls, 1992; Rhodes, 1992; Spruston et al., 1995; Hestrin et al., 1990).
- Chemicals such as nitric oxide may be released during high neural activity and gradually decay in concentration over a short time window during which learning could be enhanced (Földiák, 1992; Montague et al., 1991; Garthwaite, 2008).

The trace update rule used in the baseline simulations of VisNet (Wallis and Rolls, 1997) is equivalent to both Földiák's used in the context of translation invariance (Wallis et al., 1993) and to the earlier rule of Sutton and Barto (1981) explored in the context of modeling the temporal properties of classical conditioning, and can be summarized as follows:

(A1) $$\delta w_{j}=\alpha {\bar{y}}^{\tau }x_{j}$$

where

(A2) $${\bar{y}}^{\tau }=(1-\eta )y^{\tau }+\eta {\bar{y}}^{\tau -1}$$

and

**Table:**

*x_j_*:	*j^th^* input to the neuron.	*y*:	Output from the neuron.
*y*^τ^:	Trace value of the output of the neuron at time step τ.	α:	Learning rate.
*w_j_*:	Synaptic weight between *j^th^* input and the neuron.	η:	Trace value. The optimal value varies with presentation sequence length.

At the start of a series of investigations of different forms of the trace learning rule, Rolls and Milward (2000) demonstrated that VisNet's performance could be greatly enhanced with a modified Hebbian trace learning rule (Equation A3) that incorporated a trace of activity from the preceding time steps, with no contribution from the activity being produced by the stimulus at the current time step. This rule took the form

(A3) $$\delta w_{j}=\alpha {\bar{y}}^{\tau -1}x_{j}^{\tau }.$$

The trace shown in Equation (A3) is in the postsynaptic term. The crucial difference from the earlier rule (see Equation A1) was that the trace should be calculated up to only the preceding timestep, with no contribution to the trace from the firing on the current trial to the current stimulus. This has the effect of updating the weights based on the preceding activity of the neuron, which is likely given the spatio-temporal statistics of the visual world to be from previous transforms of the same object (Rolls and Milward, 2000; Rolls and Stringer, 2001). This is biologically not at all implausible, as considered in more detail elsewhere (Rolls, 2008, 2012), and this version of the trace rule was used in this investigation.

The optimal value of η in the trace rule is likely to be different for different layers of VisNet. For early layers with small receptive fields, few successive transforms are likely to contain similar information within the receptive field, so the value for η might be low to produce a short trace. In later layers of VisNet, successive transforms may be in the receptive field for longer, and invariance may be developing in earlier layers, so a longer trace may be beneficial. In practice, after exploration we used η values of 0.6 for layer 2, and 0.8 for layers 3 and 4. In addition, it is important to form feature combinations with high spatial precision before invariance learning supported by a temporal trace starts, in order that the feature combinations and not the individual features have invariant representations (Rolls, 2008, 2012). For this reason, purely associative learning with no temporal trace was used in layer 1 of VisNet (Rolls and Milward, 2000).

The following principled method was introduced to choose the value of the learning rate α for each layer. The mean weight change from all the neurons in that layer for each epoch of training was measured, and was set so that with slow learning over 15–50 trials, the weight changes per epoch would gradually decrease and asymptote with that number of epochs, reflecting convergence. Slow learning rates are useful in competitive nets, for if the learning rates are too high, previous learning in the synaptic weights will be overwritten by large weight changes later within the same epoch produced if a neuron starts to respond to another stimulus (Rolls, 2008). If the learning rates are too low, then no useful learning or convergence will occur. It was found that the following learning rates enabled good operation with the 100 transforms of each of 4 stimuli used in each epoch in the present investigation: Layer 1 α = 0.05; Layer 2 α = 0.03 (this is relatively high to allow for the sparse representations in layer 1); Layer 3 α = 0.005; Layer 4 α = 0.005.

To bound the growth of each neuron's synaptic weight vector, **w**_*i*_ for the *i*th neuron, its length is explicitly normalized [a method similarly employed by Malsburg (1973) which is commonly used in competitive networks (Rolls, 2008)]. An alternative, more biologically relevant implementation, using a local weight bounding operation which utilizes a form of heterosynaptic long-term depression (Rolls, 2008), has in part been explored using a version of the (Oja, 1982) rule (see Wallis and Rolls, 1997).

### A.2 The network implemented in VisNet

The network itself is designed as a series of hierarchical, convergent, competitive networks, in accordance with the hypotheses advanced above. The actual network consists of a series of four layers, constructed such that the convergence of information from the most disparate parts of the network's input layer can potentially influence firing in a single neuron in the final layer—see Figure 1. This corresponds to the scheme described by many researchers (Van Essen et al., 1992; Rolls, 1992, 2008, for example) as present in the primate visual system—see Figure 1. The forward connections to a cell in one layer are derived from a topologically related and confined region of the preceding layer. The choice of whether a connection between neurons in adjacent layers exists or not is based upon a Gaussian distribution of connection probabilities which roll off radially from the focal point of connections for each neuron. (A minor extra constraint precludes the repeated connection of any pair of cells.) In particular, the forward connections to a cell in one layer come from a small region of the preceding layer defined by the radius in Table A1 which will contain approximately 67% of the connections from the preceding layer. Table A1 shows the dimensions for the research described here, a (16×) larger version than the version of VisNet used in most of our previous investigations, which utilized 32 × 32 neurons per layer. For the research on view and translation invariance learning described here, we decreased the number of connections to layer 1 neurons to 100 (from 272), in order to increase the selectivity of the network between objects. We increased the number of connections to each neuron in layers 2–4 to 400 (from 100), because this helped layer 4 neurons to reflect evidence from neurons in previous layers about the large number of transforms (typically 100 transforms, from 4 views of each object and 25 locations) each of which corresponded to a particular object.

**Table A1:** **VisNet dimensions**.

	**Dimensions**	**# Connections**	**Radius**
Layer 4	128 × 128	400	48
Layer 3	128 × 128	400	36
Layer 2	128 × 128	400	24
Layer 1	128 × 128	100	24
Input layer	256 × 256 × 16	–	–

Figure 1 shows the general convergent network architecture used. Localization and limitation of connectivity in the network is intended to mimic cortical connectivity, partially because of the clear retention of retinal topology through regions of visual cortex. This architecture also encourages the gradual combination of features from layer to layer which has relevance to the binding problem, as described elsewhere (Rolls, 2008, 2012).

### A.3 Competition and lateral inhibition

In order to act as a competitive network some form of mutual inhibition is required within each layer, which should help to ensure that all stimuli presented are evenly represented by the neurons in each layer. This is implemented in VisNet by a form of lateral inhibition. The idea behind the lateral inhibition, apart from this being a property of cortical architecture in the brain, was to prevent too many neurons that received inputs from a similar part of the preceding layer responding to the same activity patterns. The purpose of the lateral inhibition was to ensure that different receiving neurons coded for different inputs. This is important in reducing redundancy (Rolls, 2008). The lateral inhibition is conceived as operating within a radius that was similar to that of the region within which a neuron received converging inputs from the preceding layer (because activity in one zone of topologically organized processing within a layer should not inhibit processing in another zone in the same layer, concerned perhaps with another part of the image). The lateral inhibition used in this investigation used the parameters for σ shown in **Table A3**.

The lateral inhibition and contrast enhancement just described are actually implemented in VisNet2 (Rolls and Milward, 2000) and VisNetL (Perry et al., 2010) in two stages, to produce filtering of the type illustrated elsewhere (Rolls, 2008, 2012). The lateral inhibition was implemented by convolving the activation of the neurons in a layer with a spatial filter, *I*, where δ controls the contrast and σ controls the width, and *a* and *b* index the distance away from the center of the filter

(A4) $$I_{a,b}=\{\begin{array}{ll} -\delta e^{-\frac{a^{2}+b^{2}}{\sigma^{2}}} & \text{if a}\neq \text{0 or b}\neq \text{0},\\ 1-\sum_{a\neq 0,b\neq 0}I_{a,b} & \text{if a = 0 and b = 0}. \end{array}$$

This is a filter that leaves the average activity unchanged.

The second stage involves contrast enhancement. A sigmoid activation function was used in the way described previously (Rolls and Milward, 2000):

(A5) $$y={\text{f}}^{\text{sigmoid}}(r)=\frac{1}{1+e^{-2\beta (r-\alpha )}}$$

where *r* is the activation (or firing rate) of the neuron after the lateral inhibition, *y* is the firing rate after the contrast enhancement produced by the activation function, and β is the slope or gain and α is the threshold or bias of the activation function. The sigmoid bounds the firing rate between 0 and 1 so global normalization is not required. The slope and threshold are held constant within each layer. The slope is constant throughout training, whereas the threshold is used to control the sparseness of firing rates within each layer. The (population) sparseness of the firing within a layer is defined (Rolls and Treves, 1998; Franco et al., 2007; Rolls, 2008; Rolls and Treves, 2011) as:

(A6) $$a=\frac{(\sum_{i}y_{i}/n)2^{\;}}{\sum_{i}y_{i}^{2}/n}$$

where *n* is the number of neurons in the layer. To set the sparseness to a given value, e.g., 5%, the threshold is set to the value of the 95th percentile point of the activations within the layer.

The sigmoid activation function was used with parameters (selected after a number of optimization runs) as shown in Table A2.

**Table A2:** **Sigmoid parameters for the runs with 25 locations by Rolls and Milward (2000)**.

Layer	1	2	3	4
Percentile	99.2	98	88	95
Slope β	190	40	75	26

In addition, the lateral inhibition parameters are as shown in Table A3.

**Table A3:** **Lateral inhibition parameters for the 25-location runs**.

Layer	1	2	3	4
Radius, σ	1.38	2.7	4.0	6.0
Contrast, δ	1.5	1.5	1.6	1.4

### A.4 The input to VisNet

VisNet is provided with a set of input filters which can be applied to an image to produce inputs to the network which correspond to those provided by simple cells in visual cortical area 1 (V1). The purpose of this is to enable within VisNet the more complicated response properties of cells between V1 and the inferior temporal cortex (IT) to be investigated, using as inputs natural stimuli such as those that could be applied to the retina of the real visual system. This is to facilitate comparisons between the activity of neurons in VisNet and those in the real visual system, to the same stimuli. In VisNet no attempt is made to train the response properties of simple cells, but instead we start with a defined series of filters to perform fixed feature extraction to a level equivalent to that of simple cells in V1, as have other researchers in the field (Hummel and Biederman, 1992; Buhmann et al., 1991; Fukushima, 1980), because we wish to simulate the more complicated response properties of cells between V1 and the inferior temporal cortex (IT). The elongated orientation-tuned input filters used accord with the general tuning profiles of simple cells in V1 (Hawken and Parker, 1987) and were computed by Gabor filters. Each individual filter is tuned to spatial frequency (0.0626 to 0.5 cycles / pixel over four octaves); orientation (0° to 135° in steps of 45°); and sign (±1). Of the 100 layer 1 connections, the number to each group in VisNetL is as shown in Table A4. Any zero D.C. filter can of course produce a negative as well as positive output, which would mean that this simulation of a simple cell would permit negative as well as positive firing. The response of each filter is zero thresholded and the negative results used to form a separate anti-phase input to the network. The filter outputs are also normalized across scales to compensate for the low frequency bias in the images of natural objects.

**Table A4:** **VisNet Layer 1 Connectivity**.

Frequency	0.5	0.25	0.125	0.0625
# Connections	74	19	5	2

*The frequency is in cycles per pixel*.

The Gabor filters used were similar to those used previously (Deco and Rolls, 2004). Following Daugman (1988) the receptive fields of the simple cell-like input neurons are modeled by 2D-Gabor functions. The Gabor receptive fields have five degrees of freedom given essentially by the product of an elliptical Gaussian and a complex plane wave. The first two degrees of freedom are the 2D-locations of the receptive field's center; the third is the size of the receptive field; the fourth is the orientation of the boundaries separating excitatory and inhibitory regions; and the fifth is the symmetry. This fifth degree of freedom is given in the standard Gabor transform by the real and imaginary part, i.e., by the phase of the complex function representing it, whereas in a biological context this can be done by combining pairs of neurons with even and odd receptive fields. This design is supported by the experimental work of Pollen and Ronner (1981), who found simple cells in quadrature-phase pairs. Even more, Daugman (1988) proposed that an ensemble of simple cells is best modeled as a family of 2D-Gabor wavelets sampling the frequency domain in a log-polar manner as a function of eccentricity. Experimental neurophysiological evidence constrains the relation between the free parameters that define a 2D-Gabor receptive field (De Valois and De Valois, 1988). There are three constraints fixing the relation between the width, height, orientation, and spatial frequency (Lee, 1996). The first constraint posits that the aspect ratio of the elliptical Gaussian envelope is 2:1. The second constraint postulates that the plane wave tends to have its propagating direction along the short axis of the elliptical Gaussian. The third constraint assumes that the half-amplitude bandwidth of the frequency response is about 1 to 1.5 octaves along the optimal orientation. Further, we assume that the mean is zero in order to have an admissible wavelet basis (Lee, 1996).

In more detail, the Gabor filters are constructed as follows (Deco and Rolls, 2004). We consider a pixelized grey-scale image given by a *N* × *N* matrix Γ^orig^*_ij_*. The subindices *ij* denote the spatial position of the pixel. Each pixel value is given a grey level brightness value coded in a scale between 0 (black) and 255 (white). The first step in the preprocessing consists of removing the DC component of the image (i.e., the mean value of the grey-scale intensity of the pixels). (The equivalent in the brain is the low-pass filtering performed by the retinal ganglion cells and lateral geniculate cells. The visual representation in the LGN is essentially a contrast invariant pixel representation of the image, i.e., each neuron encodes the relative brightness value at one location in visual space referred to the mean value of the image brightness.) We denote this contrast-invariant LGN representation by the *N* × *N* matrix Γ_*ij*_ defined by the equation

(A7) $$\Gamma_{ij}=\Gamma_{ij}^{\text{orig}}-\frac{1}{N^{2}}\sum_{i=1}^{N}\sum_{j=1}^{N}\Gamma_{ij}^{\text{orig}}.$$

Feedforward connections to a layer of V1 neurons perform the extraction of simple features like bars at different locations, orientations and sizes. Realistic receptive fields for V1 neurons that extract these simple features can be represented by 2D-Gabor wavelets. Lee (1996) derived a family of discretized 2D-Gabor wavelets that satisfy the wavelet theory and the neurophysiological constraints for simple cells mentioned above. They are given by an expression of the form

(A8) $$G_{pqkl}(x,y)=a^{-k}\Psi_{\Theta_{l}}(a^{-k}(x-2p),a^{-k}(y-2q))$$

where

(A9) $$\Psi_{\Theta_{l}}=\Psi (x\cos(l\Theta_{0})+y\sin(l\Theta_{0}),-x\sin(l\Theta_{0})+y\cos(l\Theta_{0})),$$

and the mother wavelet is given by

(A10) $$\Psi (x,y)=\frac{1}{\sqrt{2\pi }}e^{-\frac{1}{8}(4x^{2}+y^{2})}[e^{i\kappa x}-e^{-\frac{\kappa^{2}}{2}}].$$

In the above equations Θ_0_ = π/*L* denotes the step size of each angular rotation; *l* the index of rotation corresponding to the preferred orientation Θ_*l*_ = *l*π/*L*; *k* denotes the octave; and the indices *pq* the position of the receptive field center at *c_x_* = *p* and *c_y_* = *q*. In this form, the receptive fields at all levels cover the spatial domain in the same way, i.e., by always overlapping the receptive fields in the same fashion. In the model we use *a*=2, *b*=1 and κ=π corresponding to a spatial frequency bandwidth of one octave. We used symmetric filters with the angular spacing between the different orientations set to 45 degrees; and with 4 filter frequencies spaced one octave apart starting with 0.5 cycles per pixel, and with the sampling from the spatial frequencies set as shown in Table A4.

Cells of layer 1 receive a topologically consistent, localized, random selection of the filter responses in the input layer, under the constraint that each cell samples every filter spatial frequency and receives a constant number of inputs.

### A.5 Measures for network performance

#### A.5.1 Information theory measures

A neuron can be said to have learnt an invariant representation if it discriminates one set of stimuli from another set, across all transforms. For example, a neuron's response is translation invariant if its response to one set of stimuli irrespective of presentation is consistently higher than for all other stimuli irrespective of presentation location. Note that we state ‘set of stimuli’ since neurons in the inferior temporal cortex are not generally selective for a single stimulus but rather a subpopulation of stimuli (Baylis et al., 1985; Abbott et al., 1996; Rolls et al., 1997a; Rolls and Treves, 1998; Rolls and Deco, 2002; Franco et al., 2007; Rolls, 2007, 2008; Rolls and Treves, 2011). We used measures of network performance (Rolls and Milward, 2000) based on information theory and similar to those used in the analysis of the firing of real neurons in the brain (Rolls, 2008; Rolls and Treves, 2011). A single cell information measure was introduced which is the maximum amount of information the cell has about any one object independently of which transform (here position on the retina and view) is shown. Because the competitive algorithm used in VisNet tends to produce local representations (in which single cells become tuned to one stimulus or object), this information measure can approach log_2_ *N_S_* bits, where *N_S_* is the number of different stimuli. Indeed, it is an advantage of this measure that it has a defined maximal value, which enables how well the network is performing to be quantified. Rolls and Milward (2000) also introduced a multiple cell information measure used here, which has the advantage that it provides a measure of whether all stimuli are encoded by different neurons in the network. Again, a high value of this measure indicates good performance.

For completeness, we provide further specification of the two information theoretic measures, which are described in detail by Rolls and Milward (2000) (see Rolls, 2008 and Rolls and Treves, 2011 for an introduction to the concepts). The measures assess the extent to which either a single cell, or a population of cells, responds to the same stimulus invariantly with respect to its location, yet responds differently to different stimuli. The measures effectively show what one learns about which stimulus was presented from a single presentation of the stimulus at any randomly chosen location. Results for top (4th) layer cells are shown. High information measures thus show that cells fire similarly to the different transforms of a given stimulus (object), and differently to the other stimuli. The single cell stimulus-specific information, *I(s,R)*, is the amount of information the set of responses, *R*, has about a specific stimulus, *s* (see Rolls et al., 1997b and Rolls and Milward, 2000). *I(s,R)* is given by

(A11) $$I(s,R)=\sum_{r\in R}P(r|s)\log_{2}\frac{P(r|s)}{P(r)}$$

where *r* is an individual response from the set of responses *R* of the neuron. For each cell the performance measure used was the maximum amount of information a cell conveyed about any one stimulus. This (rather than the mutual information, *I(S,R)* where *S* is the whole set of stimuli *s*), is appropriate for a competitive network in which the cells tend to become tuned to one stimulus. (*I(s,R)* has more recently been called the stimulus-specific surprise (DeWeese and Meister, 1999; Rolls and Treves, 2011). Its average across stimuli is the mutual information *I(S,R)*.)

If all the output cells of VisNet learned to respond to the same stimulus, then the information about the set of stimuli *S* would be very poor, and would not reach its maximal value of log_2_ of the number of stimuli (in bits). The second measure that is used here is the information provided by a set of cells about the stimulus set, using the procedures described by Rolls et al. (1997a) and Rolls and Milward (2000). The multiple cell information is the mutual information between the whole set of stimuli *S* and of responses *R* calculated using a decoding procedure in which the stimulus *s*' that gave rise to the particular firing rate response vector on each trial is estimated. [The decoding step is needed because the high dimensionality of the response space would lead to an inaccurate estimate of the information if the responses were used directly, as described by Rolls et al. (1997a) and Rolls and Treves (1998).] A probability table is then constructed of the real stimuli *s* and the decoded stimuli *s*'. From this probability table, the mutual information between the set of actual stimuli *S* and the decoded estimates *S*' is calculated as

(A12) $$I(S,S^{\prime })=\sum_{s,s^{\prime }}P(s,s^{\prime })\log_{2}\frac{P(s,s^{\prime })}{P(s)P(s^{\prime })}$$

This was calculated for the subset of cells which had as single cells the most information about which stimulus was shown. In particular, in Rolls and Milward (2000) and subsequent papers, the multiple cell information was calculated from the first five cells for each stimulus that had maximal single cell information about that stimulus, that is from a population of 35 cells if there were seven stimuli (each of which might have been shown in for example 9 or 25 positions on the retina).

#### A.5.2 Pattern association decoding

The output of the inferior temporal visual cortex reaches structures such as the orbitofrontal cortex and amygdala, where associations to other stimuli are learned by a pattern association network with an associative (Hebbian) learning rule (Rolls, 2008, 2014). We therefore used a one-layer pattern association network (Rolls, 2008) to measure how well the output of VisNet could be classified into one of the objects. The pattern association network had four output neurons, one for each object. The inputs were the ten neurons from layer 4 of VisNet for each of the four objects with the best single cell information, making 40 inputs to each neuron. The network was trained with the Hebb rule:

(A13) $$\delta w_{ij}=\alpha y_{i}x_{j}$$

where δ*w_ij_* is the change of the synaptic weight *w_ij_* that results from the simultaneous (or conjunctive) presence of presynaptic firing *x_j_* and postsynaptic firing or activation *y_i_*, and α is a learning rate constant that specifies how much the synapses alter on any one pairing. The pattern associator was trained for one trial on the output of VisNet produced by every transform of each object.

Performance on the test images extracted from the scenes was tested by presenting an image to VisNet, and then measuring the classification produced by the pattern associator. Performance was measured by the percentage of the correct classifications of an image as the correct object.

This approach to measuring the performance is very biologically appropriate, for it models the type of learning thought to be implemented in structures that receive information from the inferior temporal visual cortex such as the orbitofrontal cortex and amygdala (Rolls, 2008, 2014). The small number of neurons selected from layer 4 of VisNet might correspond to the most selective for this stimulus set in a sparse distributed representation (Rolls, 2008; Rolls and Treves, 2011). The method would measure whether neurons of the type recorded in the inferior temporal visual cortex with good view and position invariance are developed in VisNet. In fact, an appropriate neuron for an input to such a decoding mechanism might have high firing rates to all or most of the view and position transforms of one of the stimuli, and smaller or no responses to any of the transforms of other objects, as found in the inferior temporal cortex for some neurons (Hasselmo et al., 1989; Perrett et al., 1991; Booth and Rolls, 1998), and as illustrated for VisNet layer 4 neuron in this investigation in Figure 5B. Moreover, it would be inappropriate to train a device such as a support vector machine or even an error correction perceptron on the outputs of all the neurons in layer 4 of VisNet to produce 4 classifications, for such learning procedures, not biologically plausible (Rolls, 2008), could map the responses produced by a multilayer network with untrained random weights to obtain good classifications.
